# Serum 25-Hydroxyvitamin D3 Levels Are Associated with Carotid Intima-Media Thickness and Carotid Atherosclerotic Plaque in Type 2 Diabetic Patients

**DOI:** 10.1155/2017/3510275

**Published:** 2017-03-28

**Authors:** Yurong Wang, Huijuan Zhang

**Affiliations:** Department of Endocrinology, The First Affiliated Hospital of Zhengzhou University, Zhengzhou 450052, China

## Abstract

*Objective.* To investigate the relationship between serum 25-hydroxyvitamin D3 [25(OH)D3] levels and carotid intima-media thickness (IMT) as well as carotid atherosclerotic plaque in patients with type 2 diabetes mellitus (T2DM). *Methods.* 314 patients with T2DM were enrolled in this study. The clinical data and laboratory examinations of subjects were recorded, such as serum 25(OH)D3, hemoglobin A1c (HbA1c), serum lipids, fasting blood glucose (FBG), and other biochemical parameters. Color Doppler ultrasound was used to measure carotid IMT and carotid atherosclerotic plaques. Patients were divided into four quartile groups according to the serum 25(OH)D3 levels from low to high: group Q1~group Q4. *Results.* From group Q1 to group Q4, carotid IMT and the incidence of plaque were gradually reduced. Serum 25(OH)D3 levels were lower in the plaque group compared with the nonplaque group (*P* < 0.01). Serum 25(OH)D3 levels were negatively correlated with the carotid IMT (*r* = −0.4, *P* < 0.01). Multiple linear stepwise regression analysis showed that serum 25(OH)D3 was independently associated with carotid IMT (*β* = −0.009, *P* < 0.01). Logistic regression analysis showed that serum 25(OH)D3 levels were independently associated with the presence of carotid plaque in T2DM (OR = 0.95; 95%CI: 0.92~0.98, *P* = 0.004). *Conclusions.* Low vitamin D status may contribute to the incidence of carotid atherosclerosis in type 2 diabetic patients.

## 1. Introduction

Vitamin D plays an important role in maintaining the integrity of bone. It contributes to the growth and development of bone in children and maintains the bone health in adults. Furthermore, vitamin D helps to prevent osteoporosis and fracture in the elderly [1]. Beyond its traditional calcium-related effects on the bone health, vitamin D is closely related to the occurrence and development of nonskeletal system diseases [2]. A large number of experimental and clinical evidence have indicated that vitamin D may be negatively correlated with autoimmune diseases [3], tumor [4], infectious disease [5], hypertension [6], T2DM [7], metabolic syndrome [8], and cardiovascular disease (CVD) [9]. Diabetic macroangiopathy is a major chronic complication and a leading cause of death in patients with T2DM, and its main pathological change is atherosclerosis. Carotid IMT is a reliable indicator of subclinical atherosclerosis [10]. There was little research about the correlation between vitamin D levels and carotid IMT in patients with T2DM, and there was no definite conclusion. In this study, we sought to assess the relationship between serum 25(OH)D3 levels and carotid IMT in patients with T2DM.

## 2. Subjects and Methods

### 2.1. Subjects

314 patients (196 males and 118 females) with T2DM who were hospitalized in the Department of Endocrinology of the First Affiliated Hospital of Zhengzhou University from September 2015 to February 2016 were enrolled. The diagnosis of the T2DM corresponds to the diagnostic criteria made by the Word Health Organization (WHO) in 1999. The exclusion criteria are as follows: patients with acute diabetic complications such as acute infection, ketoacidosis, and hyperosmolar coma; serious liver and kidney dysfunction; thyroid, parathyroid, and other endocrine gland-related diseases; autoimmune diseases; osteoporosis and other bone metabolic diseases; tumor; pregnant women; mental disease; recent surgery; taking any medications known to affect vitamin D metabolism; and patients with a recent history of excess ultraviolet (UV) ray exposure.

This study complied with the principles of the Declaration of Helsinki and was approved by the local ethics committee. Written informed consent was obtained from all subjects.

### 2.2. Research Methods

#### 2.2.1. The General Clinical Data

General clinical data including sex, age, diabetes duration, smoking history, hypertension history, family history of diabetes, height, weight, systolic blood pressure (SBP), and diastolic blood pressure (DBP) was recorded for all patients. Body mass index (BMI) was calculated as weight in kilograms divided by height per square meter.

#### 2.2.2. Laboratory Examinations

Blood samples were obtained by venipuncture after an overnight fast. Serum calcium, uric acid, triglyceride (TG), total cholesterol (TC), high-density lipoprotein cholesterol (HDL-C), low-density lipoprotein cholesterol (LDL-C), and other conventional biochemical indicators were measured on an automatic analyzer (Hitachi 7600-020, Japan). FBG was measured by glucose oxidase method. HbA1c was measured by an automated high-performance liquid chromatography analyzer. 25(OH)D3, parathyroid hormone (PTH), and osteocalcin (OSTE) were measured using an electrochemiluminescence immunoassay (Cobas e411 analyzer, Roche, Germany). The normal reference value of 25(OH)D3 levels in our laboratory was >18.0 ng/mL.

#### 2.2.3. Determination of Carotid IMT

The Aloka-*α* color Doppler diagnostic instrument with a 10–13 MHz probe was used for carotid ultrasonography by the experienced radiologists who were blind to the clinical characteristics of the patients, with subjects in the supine position. The IMT carotid was measured at three different sites on the left and right sides, and then the average of the six measurements were calculated. The carotid atherosclerotic plaque was examined as well.

### 2.3. Statistical Analysis

SPSS 21.0 statistic software (SPSS for Windows 21.0) was used to analyse all the data. Continuous variables were presented as mean ± standard deviations $\left(\overset{¯}{x}\pm s\right)$ or median (interquartile range), and categorical variables as frequency and/or percentage. An independent *t*-test was used to compare two arrays of data. Differences among multiple groups were compared with one-way ANOVA (LSD-*t*-test). Comparisons of categorical variables between/among the groups were performed with the chi-square test. Correlation analyses between two variables were performed with Pearson's correlation or Spearman's correlation. The risk factors of carotid IMT and carotid plaque were performed with multivariate linear regression analysis and logistic regression analysis. *P* values less than 0.05 were considered significant.

## 3. Results

### 3.1. Comparisons of the Clinical and Biochemical Characteristics

The range of 25(OH)D3 levels in all the subjects was (3.0–45.1) ng/mL [(14.4 ± 7.0) ng/mL, M: 13.9 ng/mL]. There were 233 patients with 25(OH)D3 levels below 18 ng/mL and 81 patients with 25(OH)D3 levels above 18 ng/mL. All the subjects were divided into four quartile groups according to the serum 25(OH)D3 levels: group Q1(<8.9 ng/mL, 71 cases); group Q2 (8.9–13.9 ng/mL, 85 cases); group Q3 (13.9–18.1 ng/mL, 77 cases); and group Q4 (≥18.1 ng/mL, 81 cases). As illustrated in Table 1, patients with lower serum 25(OH)D3 levels had a longer duration of diabetes, higher systolic blood pressure, HbA1c, LDL-C, PTH, carotid IMT, and plaque formation rate than those with higher 25(OH)D3 levels. However, the levels of serum HDL-C and calcium were lower.

Carotid IMT of patients in group Q1 [(1.23 ± 0.40) mm] and group Q2 [(1.19 ± 0.31) mm] was significantly higher than that in group Q3 [(1.06 ± 0.22) mm] and group Q4 [(0.93 ± 0.17) mm], the carotid IMT in group Q3 was significantly higher than that in group Q4 (*P* < 0.01). The incidence of carotid plaque in group Q3 (48 cases, 62.3%), group Q2 (58 cases, 68.2%), and group Q1 (40 cases, 56.3%) was higher than that in group Q4 (32 cases, 39.5%) (*P* < 0.05) (Table 1).

### 3.2. Comparisons of the Variables between the Plaque Group and the Nonplaque Group

Compared with the nonplaque group, patients in the plaque group were older, had a longer duration of diabetes, and had higher SBP, HbA1c, and LDL-C (*P* < 0.05). The number of patients with hypertension history in the plaque group was more than that in the nonplaque group (*P* < 0.05). However, the levels of serum 25(OH)D3 were significantly lower in the plaque group [178 cases, (13.4 ± 5.8) ng/mL] compared with the nonplaque group [136 cases, (15.7 ± 8.0) ng/mL] (*P* < 0.01). Other parameters were not statistically different between the two groups (Table 2).

### 3.3. Correlation Analyses between Carotid IMT and Various Variables

Pearson's correlation analyses showed that the carotid artery IMT was positively correlated with age, SBP, HbA1c, LDL-C, and diabetes duration but negatively correlated with the levels of serum 25(OH)D3 and calcium (*P* < 0.05). Spearman correlation analyses showed that the carotid artery IMT was positively correlated with smoking history and hypertension history (*P* < 0.05) (Table 3).

### 3.4. Analyses of Influence Factors of Carotid IMT and Carotid Atherosclerotic Plaque

Multivariate linear stepwise regression analysis was performed to evaluate the independent influence factors of carotid IMT. The analysis demonstrated that age (X1), HbA1c (X2), 25(OH)D3 (X3), and serum calcium (X4) were independently associated with carotid IMT. The regression equation was *Y* = 1.12 + 0.01*X*1 + 0.06*X*2 − 0.01*X*3 − 0.39*X*4. It showed that carotid IMT decreased dose dependently with increasing concentrations of 25(OH)D3. Standardized regression coefficients showed that age was the most important influence factor of carotid IMT, followed by HbA1c, 25(OH)D3, and serum calcium (Table 4).

Logistic regression analysis showed that age, diabetes duration, hypertension history, HbA1c, and LDL-C were risk factors of carotid plaque. Meanwhile, serum 25(OH)D3 was independently associated with the incidence of carotid plaques in patients with T2DM (OR = 0.95; 95%CI: 0.92~0.98, *P* = 0.004) (Table 5).

## 4. Discussion

Vitamin D3, as an essential fat-soluble vitamin, largely obtained from cutaneous exposure to ultraviolet radiation and to a lesser extent from dietary and supplements, is metabolized by the liver and then by the kidney to become 1,25-dihydroxyvitamin D3 91,25(OH)_2_D3), the tightly regulated activated molecule of vitamin D3, which can exert biological effect by combining with vitamin D receptor. However, 1,25(OH)_2_D3 has a very short half-life, so it is not generally used as a clinical biomarker to assess vitamin D status. 25(OH)D3, as the circulating storage form of vitamin D3, has a long-serum half-life, and it is easily to be detected. Accordingly, the quantitation of serum 25(OH)D3 provides a clinically useful assessment of vitamin D status [11]. In recent years, with the continuous exploration of the medical field, a growing number of studies have confirmed that vitamin D has an important impact on other system-related diseases, in addition to its classic role in calcium homeostasis and bone metabolism.

Diabetic macrovascular complications are the major causes of mortality and disability among the patients with T2DM, of which the pathological basis is atherosclerosis, and its early change is the increasing artery intima-media thickness, especially the carotid IMT. A large number of studies have demonstrated that carotid IMT can be used as a suitable surrogate marker of macroangiopathy [12]. At present, most of studies indicate that vitamin D deficiency is closely related to the incidence of diabetes. A majority of scholars hold that vitamin D affects the incidence of T2DM by affecting pancreatic beta cell function, insulin sensitivity, and systemic inflammation [13]. Several epidemiological studies have reported that vitamin D levels are inversely associated with increased arterial stiffness [14]. In the present study, serum 25(OH)D3 in patients with T2DM was at a low level, and the serum 25(OH)D3 levels in the plaque group were significantly lower than those in the nonplaque group. These findings are consistent with the above.

Our study showed that the carotid IMT levels in the lower 25(OH)D3 groups were greater than those in the higher 25(OH)D3 groups. The differences were still statistically significant after adjustment for sex, age, BMI, serum lipids, HbA1c, diabetes duration, blood pressure, smoking history, and other variables. Pearson's correlation analysis showed that the carotid artery IMT was negatively correlated with the levels of serum 25(OH)D3. Multiple linear regression analysis showed that 25(OH)D3 was independently associated with carotid IMT, after adjusting for age, diabetes duration, systolic blood pressure, HbA1c, LDL-C, and serum calcium. Logistic regression analysis showed that low serum 25(OH)D3 was the risk factor of carotid plaque. The results as described above suggested that with the decreasing of serum 25(OH)D3 levels, carotid IMT and carotid plaque showed a trend of increasing.

In addition, the correlation analyses showed that carotid IMT correlated positively to age, diabetes duration, SBP, HbA1c, LDL-C, smoking history, and hypertension history. In regression analysis, age, diabetes duration, hypertension history, HbA1c, and LDL-C were also the risk factors of carotid plaque. And the influence of these factors on carotid IMT and atherosclerosis have been confirmed in a number of previous studies. As shown in our study, patients with lower serum 25(OH)D3 levels had a longer diabetes duration, higher SBP, HbA1c, LDL-C, and lower HDL-C than those with higher 25(OH)D3 levels. These results further demonstrated that it is helpful to prevent atherosclerosis by controlling blood glucose, blood pressure, and blood lipids.

In our study, serum calcium was independently correlated with carotid IMT. Currently, there is no final conclusion of the relationship between serum calcium status and carotid IMT among type 2 diabetic individuals. A study in north Manhattan showed that subjects with no carotid plaque had lower serum calcium levels within the normal range than those with carotid plaque [15]. However, other research suggested that calcium and vitamin D supplements can help to reduce the incidence of cardiovascular disease, and the effect of vitamin D supplements was better than that of calcium supplements [16]. Further research is needed to elucidate the relationship between serum calcium status and atherosclerosis.

The following four aspects might be important for the effects of vitamin D on atherosclerosis: (1) Vitamin D can increase the expression of Ca-ATRase in vascular endothelial cells and smooth muscle cells, elevate cytosolic free calcium concentrations, induce contractile proteins expression, and then promote production of prostacyclin (PGI2), which affects the vascular tone; Vitamin D can improve endothelial function by inhibiting the inflammatory reaction and oxidative injury [17, 18], and it can decrease vascular stiffness [19]. (2) Vitamin D can improve insulin secretion/sensitivity by combing with the vitamin D receptors in pancreatic islet *β* cells and/or modulating the balance of calcium homeostasis between intracellular and extracellular. Moreover, vitamin D deficiency is associated with inflammation which has been established as a key pathogenic mechanism in atherosclerosis. (3) Vitamin D can decrease the activity of renin angiotensin aldosterone system (RAAS), which subsequently reduces blood pressure and decreases the risk of atherosclerosis. (4) It is well known that atherosclerosis is associated with dyslipidemia. LDL-C is a recognized risk factor for atherosclerosis, but HDL-C has an antagonistic effect on atherosclerosis. Several studies have suggested that dyslipidemia is associated with vitamin D deficiency. Patients with vitamin D deficiency had higher LDL-C and lower HDL-C levels [20].

## 5. Conclusions

In conclusion, serum 25(OH)D3 was negatively correlated with carotid IMT and the incidence of carotid plaques in patients with T2DM. When serum 25(OH)D3 concentration is below a certain level, the incidence of atherosclerosis in type 2 diabetic patients may increase. Vitamin D deficiency may predict subclinical atherosclerosis in diabetic patients, and vitamin D supplementation might be a clinical intervention to prevent atherosclerosis in patients with T2DM. Remarkably, our study is a cross-sectional one. It is necessary to carry out further follow-up and researches.

## Figures and Tables

**Table 1:** Baseline characteristics of type 2 diabetic patients by quartiles of serum 25(OH)D3 concentrations.

Variables	Q1 (<8.9 ng/mL)	Q2 (8.9–13.9 ng/mL)	Q3 (13.9–18.1 ng/mL)	Q4 (≥18.1 ng/mL)	*P* value
*n*	71	85	77	81	—
Sex (M/F)	40/31	51/34	50/27	55/26	0.462
Age (years)	53.0 ± 10.5	53.2 ± 12.4	52.4 ± 12.4	50.9 ± 12.4	0.614
BMI (kg/m^2^)	25.8 ± 3.7	25.3 ± 4.1	25.5 ± 2.9	25.0 ± 3.4	0.086
SBP (mmHg)	138.8 ± 20.2	136.8 ± 14.3	131.5 ± 17.5^a^	133.5 ± 18.2	0.052
DBP (mmHg)	83.2 ± 11.4	85.5 ± 10.3	82.6 ± 8.6	83.0 ± 10.3	0.235
Total cholesterol (mmol/L)	4.5 ± 1.1	4.4 ± 1.1	4.2 ± 0.9	4.4 ± 1.0	0.300
Triglyceride (mmol/L)	2.2 ± 1.7	1.9 ± 1.8	1.9 ± 1.3	1.9 ± 1.4	0.485
HDL-C (mmol/L)	1.1 ± 0.3	1.2 ± 0.4^a^	1.1 ± 0.2	1.1 ± 0.2	0.064
LDL-C (mmol/L)	3.0 ± 0.8	2.8 ± 0.9	2.6 ± 0.8^a^	2.6 ± 0.8^a^	0.028
Uric acid (μmol/L)	284.6 ± 91.1	266.5 ± 76.8	284.1 ± 92.7	275.3 ± 80.0	0.484
Parathyroid hormone (pg/mL)	47.8 ± 36.3	38.0 ± 15.0^ab^	37.2 ± 12.4^a^	31.3 ± 9.8^a^	0.000
Osteocalcin (ng/mL)	13.4 ± 5.7	13.9 ± 5.1	12.8 ± 6.1	13.9 ± 5.3	0.665
FBG (mmol/L)	8.0 ± 3.4	7.9 ± 2.7	7.9 ± 2.8	8.7 ± 3.2	0.318
HbA1c (%)	10.2 ± 1.2	10.0 ± 1.3^b^	9.6 ± 1.6^ab^	8.6 ± 1.4^a^	0.000
Diabetes duration (months)	112.5 ± 87.9	98.0 ± 86.6	84.8 ± 71.0^a^	82.3 ± 75.4^a^	0.085
Calcium (mmol/L)	2.22 ± 0.13	2.31 ± 0.10^a^	2.32 ± 0.10^a^	2.31 ± 0.10^a^	0.000
Carotid IMT (mm)	1.23 ± 0.40	1.19 ± 0.31^bc^	1.06 ± 0.22^ab^	0.93 ± 0.17^a^	0.000
Carotid plaque [*n* (%)]	40 (56.3%)^b^	58 (68.2%)^b^	48 (62.3%)^b^	32 (39.5%)	0.002
Smoking history [*n* (%)]	27 (38.0%)	29 (34.1%)	22 (28.6%)	24 (29.6%)	0.586
Family history of DM [*n* (%)]	37 (52.1%)	39 (45.9%)	30 (39.0%)	44 (54.3%)	0.217
Hypertension history [*n* (%)]	30 (42.3%)	39 (45.9%)	31 (40.3%)	29 (35.8%)	0.614

Data are presented as mean ± SD for continuous variables and number (percentages) for dichotomous variables. Differences were assessed by the LSD-*t*-test (for continuous variables) and by the chi-square test (for categorical variables). 1 mmHg = 0.133 kPa; SBP: systolic blood pressure; DBP: diastolic blood pressure; HDL-C: high-density lipoprotein cholesterol; LDL-C: low-density lipoprotein cholesterol; FBG: fasting blood glucose; HbA1c: glycosylated hemoglobin c; carotid IMT: carotid intima-media thickness; ^a^analysis of variance with LSD-*t*-test or chi-square test: *P* < 0.05 versus group Q1; ^b^analysis of variance with LSD-*t*-test or chi-square test: *P* < 0.05 versus group Q4; ^c^analysis of variance with LSD-*t*-test or chi-square test: *P* < 0.05 versus group Q3.

**Table 2:** Comparison of clinical characteristics in type 2 diabetic patients with and without carotid plaque.

Variables	Plaque group (*n* = 178)	Nonplaque group (*n* = 136)	*P* value
Sex (M/F)	118/60	78/58	0.126
Age (years)^∗^	56.5 ± 10.8	47.0 ± 11.3	0.000
BMI (kg/m2)	25.2 ± 3.4	25.5 ± 3.7	0.603
SBP (mmHg)	136.8 ± 18.3	132.9 ± 16.7	0.055
DBP (mmHg)	82.8 ± 9.9	84.6 ± 10.5	0.127
Total cholesterol (mmol/L)	4.4 ± 1.1	4.3 ± 1.0	0.720
Triglyceride (mmol/L)	1.9 ± 1.5	2.0 ± 1.6	0.531
HDL-C (mmol/L)	1.1 ± 0.3	1.1 ± 0.3	0.451
LDL-C (mmol/L)^∗^	2.8 ± 0.9	2.6 ± 0.7	0.010
Uric acid (*μ*mol/L)	273.7 ± 82.4	281.7 ± 88.3	0.344
25(OH)D3 (ng/mL)^∗^	13.4 ± 5.8	15.7 ± 8.0	0.004
Parathyroid hormone (pg/mL)	38.7 ± 23.1	37.7 ± 18.2	0.695
Osteocalcin (ng/mL)	13.1 ± 6.1	14.1 ± 4.7	0.199
FBG (mmol/L)	8.2 ± 3.2	8.0 ± 2.9	0.483
HbA1c (%)^∗^	10.1 ± 1.5	8.9 ± 1.3	0.000
Diabetes duration (months)^∗^	113.7 ± 85.5	68.2 ± 66.6	0.000
Calcium (mmol/L)	2.3 ± 0.1	2.3 ± 0.1	0.197
Carotid IMT (mm)^∗^	1.3 ± 0.3	0.9 ± 0.1	0.000
Smoking history [*n* (%)]	65 (36.5%)	37 (27.2%)	0.089
Family history of DM [*n* (%)]	84 (47.2%)	66 (48.5%)	0.821
Hypertension history [*n* (%)]^∗^	86 (48.3%)	43 (31.6%)	0.004

Differences were assessed by the independent sample *t*-test (for continuous variables) and by the chi-square test (for categorical variables). ∗ indicates that the comparisons of variables between the two groups were statistically significant, *P* < 0.05.

**Table 3:** Correlation analysis: correlative factors of carotid IMT.

Variables	*r*	*P* value
Age	0.4	0.000
Diabetes duration	0.3	0.000
SBP	0.2	0.001
HbA1c	0.4	0.000
LDL-C	0.1	0.047
25(OH)D3	−0.4	0.000
Calcium	−0.3	0.000
Smoking history	0.1	0.037
Hypertension history	0.2	0.001

**Table 4:** Multiple linear regression analysis: independent influence factors of carotid artery IMT.

Independent factors	Unstandardized coefficient		*t*	*P* value	Standardized coefficients Beta
	*β*	Std. error			
HbA1c	0.06	0.010	5.42	0.000	0.27
Age	−0.01	0.001	7.84	0.000	0.36
25(OH)D3	0.01	0.002	−3.93	0.000	−0.20
Calcium	−0.39	0.123	−3.20	0.002	−0.15
Constant	1.12	0.306	0.65	0.000	—

In this model, BMI, diabetes duration, SBP, and LDL-C were also included as covariates, but they were not independently associated with carotid IMT.

**Table 5:** Logistic regression analysis: risk factor of carotid atherosclerotic plaque.

Risk factors	*β*	OR	95%CI	*P* value
Age	0.08	1.08	1.06–1.11	0.000
HbA1c	0.72	2.06	1.65–2.56	0.000
LDL-C	0.36	1.43	1.08–1.90	0.013
25(OH)D3	−0.05	0.95	0.92–0.98	0.004
Diabetes duration	0.01	1.01	1.01–1.01	0.000
Hypertension history	0.70	2.02	1.27–3.22	0.003
